# Bacterial Lipodepsipeptides and Some of Their Derivatives and Cyclic Dipeptides as Potential Agents for Biocontrol of Pathogenic Bacteria and Fungi of Agrarian Plants

**DOI:** 10.1021/acs.jafc.1c08139

**Published:** 2022-04-08

**Authors:** Stefany Castaldi, Alessio Cimmino, Marco Masi, Antonio Evidente

**Affiliations:** † Department of Biology, University of Naples Federico II, Complesso Universitario Monte S. Angelo, 80126 Napoli, Italy; ‡ Department of Chemical Sciences, University of Naples Federico II, Complesso Universitario Monte S. Angelo, 80126 Napoli, Italy

**Keywords:** agrarian plant diseases, bacterial and fungal pathogens, biopesticides, lipodepsipeptides, cyclic dipeptides, antimicrobial activity

## Abstract

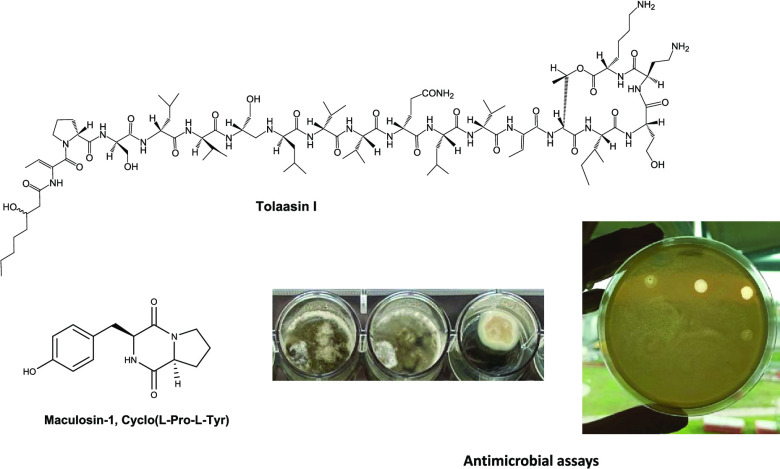

Biotic stresses (fungi, bacteria, insects, weeds, etc.) are some of the most important causes of the decrease in the quality and quantity of crops that could become an emergency due to a noteworthy increase in the world population. Thus, to overcome these problems, massive use of chemical pesticides has been carried out with heavy consequences for environmental pollution and food safety. An eco-friendly alternative can be using natural compound-based biopesticides with high efficacy and selectivity. Some bacterial lipodepsipeptides (tolaasins I, II, A, D, and E and WLIP together with hexacetyl- and tetrahydro-tolaasin I and WLIP methyl ester) and cyclic dipeptides (cyclo(l-Pro-l-Tyr), cyclo(d-Pro-l-Tyr), cyclo(l-Pro-l-Val), and cyclo(l-Pro-l-Leu)) were assayed against several pathogenic bacteria and fungi of important agrarian plants. Lipodepsipeptides showed strong growth inhibition of all microorganisms tested in the range of 0.1–0.8 μg/mL, while cyclodipeptides, despite preserving this ability, showed a noteworthily reduced antimicrobial activity being active only in the range of 15–900 μg/mL. Among the lipodepsipeptides and cyclic dipeptides assayed, tolaasin d and cyclo(l-Pro-l-Tyr) (also named maculosin-1) appeared to be the most toxic compounds. Some structure–activity relationships of lipodepsipeptides were also discussed along with their practical application as biopesticides in agriculture.

## Introduction

People, since ancient times, had worldwide developed agriculture as the first human activity to produce food in high quantity and quality. This necessity increased over time for a constant increase in the world population, which will be almost 10 billion by 2050.^1,2^ These aspects, despite the noteworthy technological progress done in agriculture, are becoming an emergency due to the strong reduction of natural sources, the environmental pollution, and climate changes.^3,4^ Biotic stresses, including microbial pathogens, weeds, insects, etc., represent the main causes of severe losses in agrarian production and food safety. Up to this day, control of these damaging agents has been done with the massive use of synthetic pesticides. The latter can cause environmental pollution, induce resistance in the host plants, and are responsible for the presence of toxic residues in agricultural products.^5,6^ These problems prompted efforts to develop integrated pest management^5^ to reduce or eliminate synthetic pesticides significantly. A valid and efficacy alternative is represented by biopesticides, which are easy degradable and represent no risk for human and animal health as strongly required by consumers and by public administrators.^3,7^

Natural products are the most important source for finding substances with different biological activities, new carbon skeletons to overcome resistance phenomena, and potential applications as new eco-friendly solutions in various fields.^6,8^

Among these classes of natural bioactive metabolites, there are lipodepsipeptides and cyclic dipeptides. Lipodepsipeptides are biologically active metabolites produced by different bacteria and are constituted by three moieties: (i) a macrocyclic peptide lactone; (ii) a linear peptide; and (iii) fatty acid. These lipodepsipeptides, containing unusual amino acids also with an opposite stereochemistry, are classified according to their primary structures into two groups. Syringotoxins, syringomycins, pseudomycins, and syringostatins belong to the first group. Those containing from 18 to 25 amino acid residues, most of which have a d-stereochemistry, such as syringopeptins, fuscopeptins, tolaasins, and corpeptins, are reported in the second group.^9^ In the latter one, the C-terminal group forms a lactone ring constituting from 5 (corpeptins, tolaasins, and fuscopeptins) to 8 (syringopeptins) amino acids. The first reported nonapeptides were syringomycins, a subgroup synthesized by the plant pathogenic bacterium *Pseudomonas syringae* pv. *syringae* showing antifungal activities. They targeted the fungal plasma membrane, and some studies on their mode of action were also performed.^10^ Successively, the other nonapeptides syringostatins, syringotoxins, and pseudomicines were produced by *P. syringae* pv. *syringae* but isolated from different infected host plants.^11^ Other lipodepsipeptides such as syringopeptines,^12^ fuscopetines,^13^ and corpeptines^14,15^ were produced by *P. syringae* pv. *syringae*, *Pseudomonas fuscovaginae*, *Pseudomonas corrugata*, and *Pseudomonas cichorii*. Lipodepsipeptides in addition to phytotoxic and antifungal activities also showed potential antibiotic activity and thus potential against the bacterial species that have developed resistance to common antibiotics.^16^

**Figure 1:**
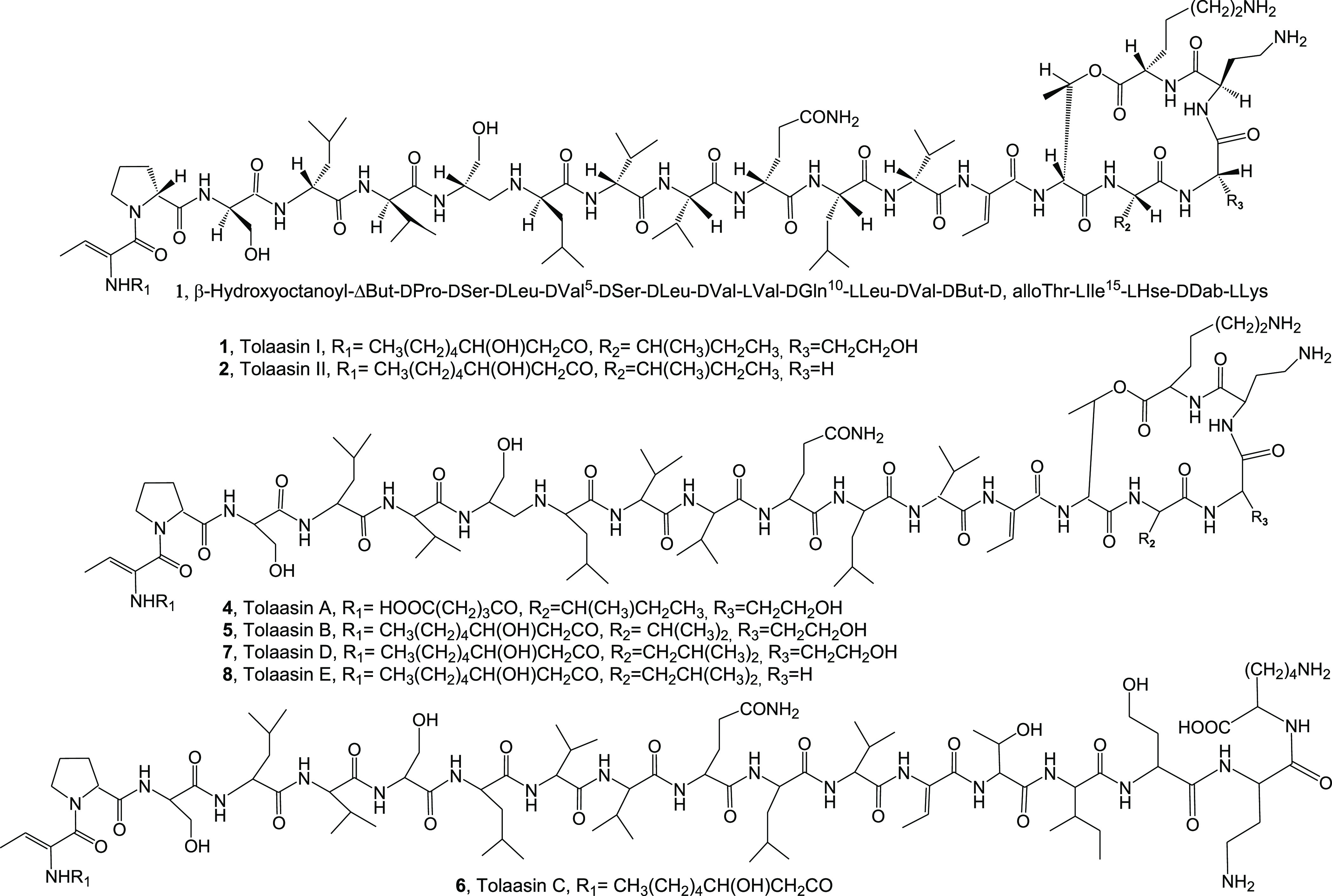
Structures of tolaasins I, II, A-E (**1**, **2**, **4**–**8**).

**Figure 2:**
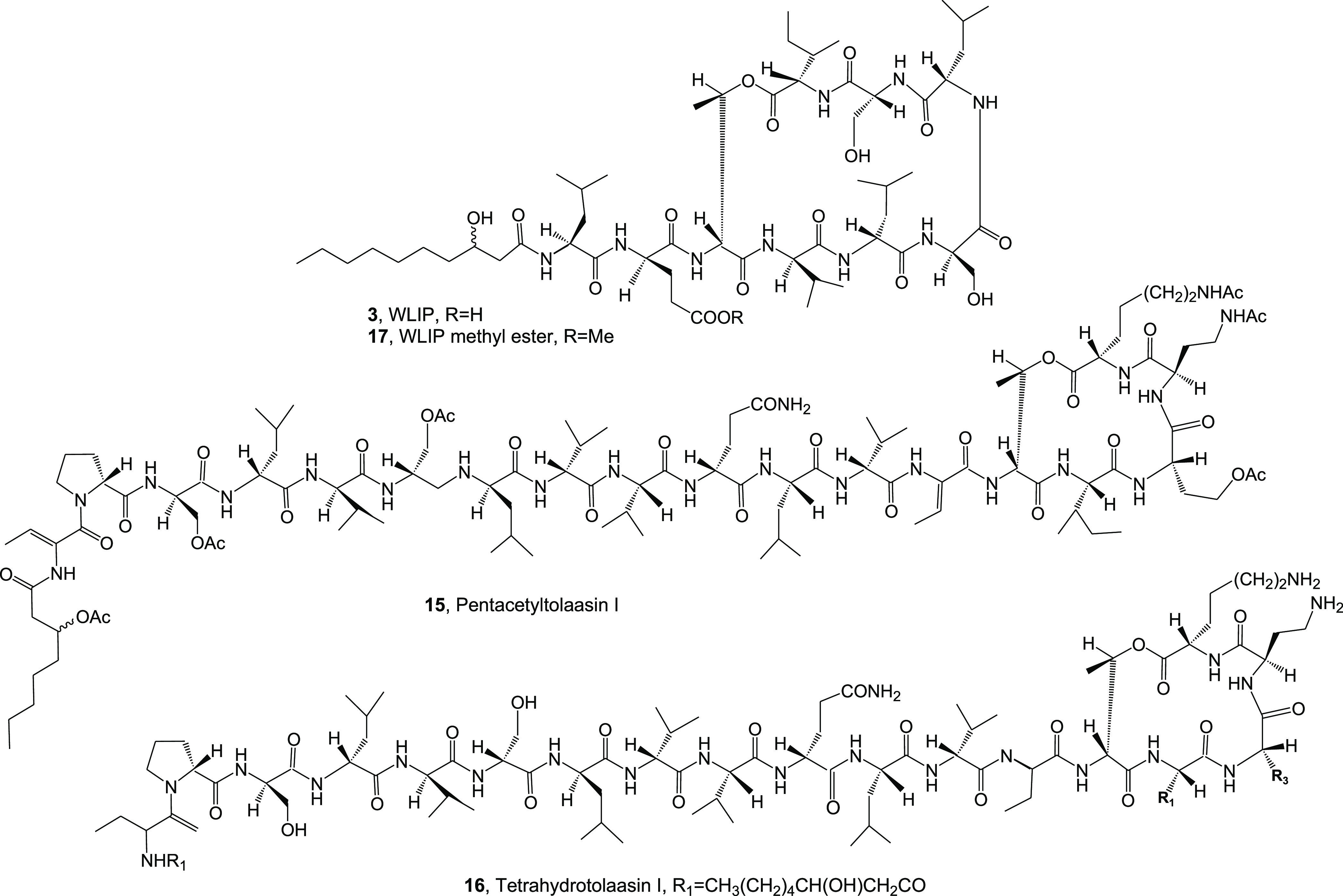
Structures of WLIP, hexacetyl- and tatrahydro-tolaasin I, and methyl ester of WLIP (**3, 15–17**).

Also, pathogenic bacteria of cultivated mushroom produce lipodepsipeptides with different biological activities such as *Burkholderia gladioli* pv. *agaricicola*, *Pseudomonas tolaasii*, and *Pseudomonas reactans*. The main bioactive lipodepsipeptides produced by both *Pseudomonas* strains are tolaasins I and II (**1** and **2**, Figure 1), which themselves differed in the substitution of the homoserine residue (Hse16) of macrocyclic lactone with a glycine residue,^17^ and the so-called white line inducing principle (WLIP, **3**, Figure 2).^18^ The role that these metabolites play in the diseases and their biological activities were extensively studied.^19^*P. tolaasii*, pathogen of *Agaricus bisporus* and *Pleurotus ostreatus*, also showed to produce, despite being in lesser amounts, other tolaasins named tolaasins A, B, C, D, and E (**4**–**8**, Figure 1). They differed from tolaasins I and II in the peptide chain, as observed in other lipodepsipeptides of bacterial origin, and preserved the β-hydroxyoctanoyl ϕ group at the *N*-terminus, except for tolaasin A, in which the acyl moiety was a γ-carboxybutanoyl ϕ chain. When tested on fungi, yeast, and bacteria, they showed antimicrobial activity against Gram-positive bacteria, which appeared to be the most sensitive, and this activity seemed to be related to the structural differences of the analogues.^20^

The close naturally occurring cyclopeptides exhibit potent biological activities, including insecticidal, antimicrobial, antifungal, and antiproliferative. They are produced by marine organisms^21^ and plants.^22^ A subgroup of this class of natural compounds is the cyclodipeptides, also known as 2,5-diketopiperazines, which showed various biological activities and displayed strong resistance against enzymatic hydrolysis, thus attracting great interest in a variety of fields spanning from functional materials to drug discovery.^23^

**Figure 3:**
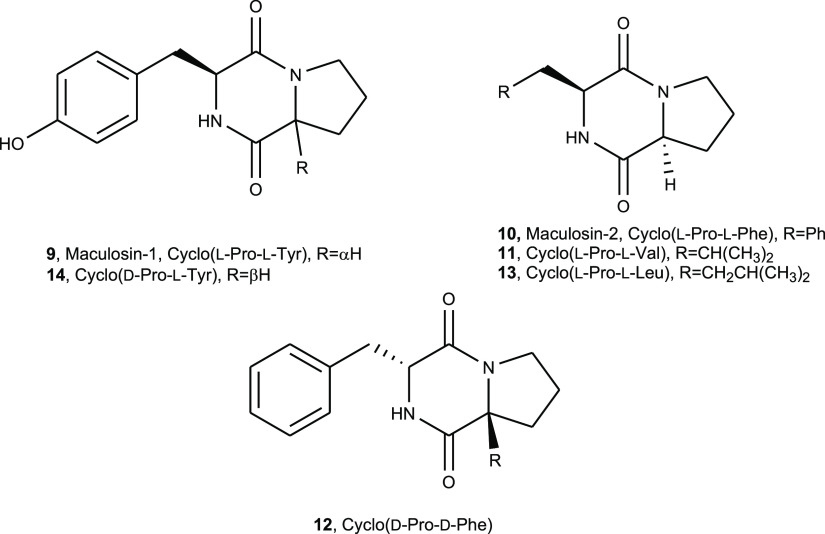
Structures of 2,5-diketopiperazine maculosin-1 (cyclo(l-Pro-l-Tyr), maculosin-2 (cyclo(l-Pro-l-Phe)), cyclo(l-Pro-l-Val), cyclo(d-Pro-d-Phe), cyclo(l-Pro-l-Leu), and cyclo(d-Pro-l-Tyr) (**9**–**14**)).

Among 2,5-diketopiperazines, the most known is maculosin-1 (cyclo(l-Pro-l-Tyr)) (**9**, Figure 3). Compound **9** is a host-specific phytotoxin produced by *Alternaria alternata*, a pathogen of knapweed.^24^ The same fungus also synthesizes cyclo(l-Pro-l-Phe) (maculosin-2) and cyclo(Pro-Ala), cyclo(Pro-Val), cyclo(Pro-Hle), cyclo(Pro-Leu), and cyclo(l-Pro-d-Phe), as potential biocontrol agents of knapweed.^24^

Compound **9** was also recently isolated from *Lysobacter capsici* AZ78 and showed antifungal activity against *Phytophthora infestans* and *Plasmopara viticola*, two pathogens of important crops.^25^ Some derivatives of maculosin-1 were also prepared and their antifungal activity, compared to those of the parent compound (**9**) and maculosin-2 cyclo(l-Pro-l-Phe) (**10**, Figure 3), was tested against *P. infestans*. Among them, the azido derivative of **9** showed strong antifungal activity, suggesting its potential use as a biofungicide.^26^ To corroborate these results, **9** was applied on tomato leaves to prevent the occurrence of late blight lesions.^25^ These results prompted an in-depth investigation of 2,5-diketopiperazine production by *L. capsici*. In fact, cyclo(l-Pro-l-Val), cyclo(d-Pro-d-Phe), cyclo(l-Pro-l-Leu), and cyclo(d-Pro-l-Tyr) (**11**–**14**, Figure 3) were successively isolated from the same bacterial cultures and were tested together with maculosins-1 and 2 (**9** and **10**) against the phytopathogenic Gram-positive bacterium *Rhodococcus fascians* LMG.^27^ Among all the 2,5-diketopiperazines assayed, compound **11** showed toxicity similar to that of chloramphenicol, a positive control, when used at the same concentration. These results and reported data suggest that 2,5-diketopiperazines could be proposed as potential biopesticides due to their broad activity spectrum against phytopathogenic microorganisms.

Thus, this article reports the antimicrobial activity of five lipodepsipeptides (**1**, **2**, **7**, and **8**), WLIP (**3**), two tolaasin I derivatives (**15** and **16**), one WLIP derivative (**17**), and four diketopiperazines (**9**, **11**, **13**, and **14**) against several pathogenic bacteria and fungi of agrarian plants. Results of structure–activity relationships were also discussed.

## Materials and Methods

### General Experimental Procedures

Optical rotation, ^1^H NMR spectra, electrospray ionization mass spectrometry (ESI MS) analysis, analytical and preparative thin-layer chromatography (TLC), and column chromatography were performed as previously reported.^20,25^ Reverse-phase high-performance liquid chromatography (HPLC) of the tolaasin crude mixture was performed as previously reported.^20^

### Production and Purification of Tolaasins from *P. tolaasii*

Tolaasins I, II, D, and E were produced growing *P. tolaasii* (strain type NCPPB2192) in liquid King’s B medium stirred culture at 25 °C as previously reported.^20^ The culture was centrifuged and lyophilized, and tolaasins were purified from the culture filtrates (1.350 L) according to a previously reported method.^28^ Briefly, after acidification of the culture filtrates, the precipitate was discarded, and tolaasins were precipitated by adding CaCl_2_. After more steps of washing with small volumes of MeOH followed by washing with small volume of Milli-Q water, the crude residue was desalted by G-10 column chromatography, and the tolaasin-containing fractions were combined and lyophilized to give a white solid residue. The tolaasin mixture (67.6 mg) was purified by HPLC using a reverse-phase semipreparative column eluted with a gradient MeCN-0.1% TFA and afforded tolaasins I, II, D, and E (**1**, **2**, **7**, and **8**). This procedure was repeated more times to accumulate the tolaasins in discrete amounts. Tolaasins I, II, D, and E were identified by ^1^H NMR and ESIMS spectra in comparison with those of standard samples. Their purity was >98% as ascertained by HPLC analysis.

### Acetylation of Tolaasin I

Tolaasin I (**1**, 2.0 mg) was dissolved in dry pyridine (100 μL) and acetylated with AC_2_O (100 μL). The reaction was carried out at room temperature overnight and stopped by adding MeOH. Pyridine was eliminated under a N_2_ stream of the azeotrope formed by addition of C_6_H_6_. The organic residue was purified with TLC using *i*-PrOH/H_2_O (8/2, v/v) as an eluent, affording the hexacetyl derivative of tolaasin I (**15**, 1.9 mg, 85%) as an amorphous solid. Compound **15** had a ^1^H NMR spectrum (Figure S2, SI) that essentially differs from that of tolassin I (Figure S1, SI), recorded under the same conditions for the presence of the singlets of five acetyl groups in the range of δ 2.2–1.99; ESI MS (+), *m*/*z*: 2260 [M + Na]^+^ (Figure S3, SI).

### Hydrogenation of Tolaasin I

Tolaasin I (**1**, 2.7 mg) was dissolved in MeOH (1 mL) and added to a suspension of 95% PtO_2_/C in MeOH (1 mL) presaturated with H_2_ gas for 30 min under stirring. The reaction was performed with H_2_ at atmospheric pressure at room temperature under stirring in the dark. The reaction was completed after 24 h and stopped by filtration of the catalyst. The solution was evaporated under reduced pressure to give the tetrahydroderivative of tolaasin I (**16**, 2.6 mg, 96%) as an amorphous solid. The ^1^H NMR spectrum recorded in CD_3_OD (Figure S4, SI) essentially differed from that of tolaasin recorded under the same conditions (Figure S1, SI) for the absence of olefinic protons; ESIMS (+) *m*/*z*: 1990 [M + H]^+^ (Figure S5, SI).

### Production and Purification of WLIP

*P. reactans* NCPPB1311 was grown on liquid KB medium at 25 °C under shaking, as previously reported.^29^ Briefly, the lyophilized culture filtrate (1.4 L) was dissolved in MIlli-Q water (1.3 L) and centrifuged at 10 000 rpm at 15 °C for 30 min. The supernantant was filtered on a Wathman n. 42 paper disk, acidified up to pH 5 with 1 N HCl, and left at room temperature overnight. The precipitate dissolved in Millli-Q water was alkalinized up to pH 7.5 with 1 N NaOH, and the solution was filtered on Whatman n. 40 paper disks. It was acidified up to pH 5 with 1N HCl. The precipitate was collected by centrifugation at 10 000 rpm at 15 °C for 30 min, oven-dried at 50 °C, and then dissolved in MeOH (100 mL). The suspension was then filtered on Whatman n. 42 paper disks, and the filtrate was evaporated under vacuum. The solid residue was washed with MeOH (10 mL), centrifuged at 8000 rpm at 15 °C for 30 min, then dissolved in MeOH (100 mL), and dried under vacuum to give crude WLIP (250 mg). The latter crystallized as white needles (216 mg) with blowing in water vapor, according to the procedure reported by Mortishire-Smith (1991).^18^ WLIP was identified by ^1^H NMR and ESIMS spectra in comparison with those of standard samples. Its purity was >98% as ascertained by HPLC analysis.

### WLIP Methyl Ester

A solution of WLIP (**3**, 15.1 mg) was dissolved MeOH (1 mL), and an ethereal solution of diazomethane was added up to a yellow persistent color. The reaction was performed at 0 °C for 48 h and stopped by evaporation of the solution using reduced pressure. The residue was purified by TLC eluted with EtOAc/MeOH/H_2_O (85/20/10), yielding the methyl ester of WLIP (**17**, 14.1 mg, 93%) as an amorphous solid. Compound **17** had a ^1^H NMR spectrum (Figure S6, SI) that essentially differed from that of WLIP (Figure S7, SI) for the presence of the singlet at δ 3.70 due to the ester methyl group; ESIMS (+) *m*/*z*: 1289 [M + Na]^+^, 1275 [M + H]^+^ (Figure S8, SI).

### Production and Purification of 2,5-Diketopiperazines

*L. capsici* AZ78 cultures were obtained as previously described.^30^ The lyophilized culture filtrates (20 L) were dissolved in Milli-Q water (2 L) and extracted with EtOAc (3 × 2 L). The corresponding extract was fractionated according the procedure previously reported.^27^ In particular, the organic extracts were combined and dried under vacuum to give a solid residue (1.56 g). The latter was chromatographed on a silica gel eluted with CHCl_3_/*i*-PrOH (9/1) and then with CHCl_3_/*i*-PrOH (7/3), yielding 10 groups of homogeneous fractions (F1–F10). The F2 residue (302 mg) was subjected to another fractionation by column chromatography, using CHCl_3_/*i*-PrOH (9/1) as an eluent. A total of 10 groups of homogeneous fractions were collected (F2.1–F2.10). The F2.3 residue (13.9 mg) appeared to be a pure metabolite, identified as cyclo(l-Pro-l-Val) (**11**). The F2.4 residue (51.3 mg) was further purified by TLC, eluted with CHCl_3_/*i*-PrOH (9/1), yielding four groups of homogeneous fractions (F2.4.1–F2.4.4). The F2.4.2 residue (36.2 mg) was further purified by more steps of TLC, giving further amounts of **11** (4 mg) and cyclo(l-Pro-l-Leu) (**13**, 3.7 mg). The residue (62.5 mg) of F3 was further purified by several steps of TLC, yielding further amounts of **11** (5.5 mg), maculosin-1 (cyclo(l-Pro-l-Tyr) (**9**, 11.9 mg), and cyclo(d-Pro-l-Tyr) (**14**, 18.4 mg)). Their identity was ascertained by ^1^H NMR and ESI MS spectra in comparison with those of standards. Their purity was >98% as ascertained by HPLC analysis.

### Minimum Inhibitory Concentrations (MIC)

#### Antimicrobial Assay

The antimicrobial assay was carried out as described in Bassarello et al. (2004)^20^ with some modifications. Bacteria were grown in LB broth at 25 or 37 °C overnight at 150 rpm. A total of 500 μL of a suspension containing about 10^8^ cfu mL^–1^ were added to 3 mL of LB soft agar (0.7%) and poured onto plates containing 7 mL of LB broth with agar 1.8%. After agar gelification, 10 μL drops of serial dilutions of different lipodepsipeptides and their derivatives (from 0.1 to 1 μg/mL) and cyclic dipeptides (from 10 to 1000 μg/mL) were tested. After 24 ± 48 h of incubation at 25 or 37 °C, the end serial dilution inhibiting the growth of the bacteria in the area of application of 10 μL solutions was recorded. The plates containing the bacteria alone were used as a control. The experiment was performed in triplicate.

#### Antifungal Assay

The antifungal activity was performed in 24-well culture plates according to the method previously described^31^ with some modification. Serial dilutions of different lipodepsipeptides (from 0.1 to 1 μg/mL) and cyclic dipeptides (from 10 to 1000 μg/mL) were dissolved in a volume of 500 μL of ultrapure Milli-Q and finally inoculated with 500 μL of 2× potato dextrose broth (Difco) containing the *Colletotrichum truncatum* plug of 4 mm × 4 mm diameter. As a control, *C. truncatum* plugs (4 mm × 4 mm) were grown in 2× PD broth diluted with 500 μL of ultrapure Milli-Q water, and the plates were incubated at 28 °C for 7 days. The MIC was measured as the lowest concentration of antifungal agent at which there was no visible growth of the fungus after incubation. The experiment was performed in triplicate.

## Results and Discussion

Bacteria belonging to the *Pseudomonas* genus were used in this study; all are causal agents of severe diseases of important agrarian plants. Among them, there is *Burkholderia caryophylli* (syn. *Pseudomonas caryophylli*) responsible for bacterial wilt of carnation resulting in serious losses in carnation production.^32^ From its culture filtrates were isolated three polyunsaturated C:17 fatty acids and other three metabolites; the latter were obtained as an interconvertible mixture and named caryoynencins A-C. The latter showed strong antibacterial activity against Gram-positive and Gram-negative bacteria such as *Staphylococcus aureus*, *Bacillus subtilis*, *Escherichia coli*, and *Klabsiella pneumoniae* but had no phytotoxicity.^33^ Although the culture filtrates exhibited phytotoxicity toward the host and nonhost plants, up to a day, phytotoxins were not isolated from *B. caryophylli*, but from some preliminary experiments carried out by some of the authors, they should be lipodepsipeptides (private communication). Extensive work was done by some of the authors on the lipopolysaccharides (LPS) present in the outer membrane of this bacterium as in general, LPS plays an important role in the first process of pathogenesis and in particular in the interaction of the plant and pathogen.^34^ The LPSs of *P. caryophylli* appeared to be constituted by two homopolysaccharide chains, with the major one built up of (1 → 7)-linked α-cryophyllose [3,6,10-trideoxy-4-*C*-(d-glycero-1-hydroxyethyl)-d-erythro-d-gulo-decose] residues and the minor one made up of (1 → 7)-linked β-caryose (4,8-cyclo-3,9-dideoxy-l-erythro-d-ido-nonose) residues. A third polysaccharide fraction mainly constituted by heptose and glucose was also isolated.^35^ The main polysaccharide, named caryophyllane, was constituted by a repeating unit of a novel 4-branched monosaccharide, named caryophyllose, characterized as trideoxy-*C*-[(*R*)-1-hydroxyethyl]-d-erythro-d-gulo-decose.^36,37^ The minor polysaccharide, named carian, was constituted by the repeating unit of new cyclic monosccharide, named caryose, characterized as carbocyclic (4,8-cyclo-3,9-dideoxy-l-erythro-d-*ido*-nonose).^38^ Another bacterium used is *P. syringae* pv. *panici*, a worldwide diffused pathogen, which induces diseases in different plants including crops such as rice, lilac, millet, and pearl millet.^39^ In rice, *P. syringae* pv. *panici* induces brown stripe disease.^40^*Pseudomonassyringae* pv. *tabaci* was also included among the bacteria used in this study as it induces brown spots on tobacco, a disease named wildfire, with severe economic consequences.^41^ The same is for *P. syringae* pv. *siringae* (Pss), which is the most polyphagous bacterium in the *P. syringae* complex due to its wide host range, first affecting woody and herbaceous host plants. In early 1990s, Pss caused apical necrosis of mango trees, a severe disease in Southern Spain. A lot of studies had been carried out on this pathogen, whose results are reported in some reviews as that published by Gutiérrez-Barranquero et al.^42^*Pseudomonassyringae* pv. *japonica*, also included in the bacteria tested, induced the black node disease of barley (*Hordeum vulgare* L.) and wheat (*Triticum aestiuum* L.) and was initially classified as *Pseudomonas striafaciens* var. *japonica*.^43^ The other three bacteria tested were *B. subtilis*, *Bacillus megaterium*, and *E. coli*, which are laboratory strains. *Colletotrichum truncatum* was selected, among the phytopathogenic fungi available, as the only strain to test because very low amounts of both lipodespsipeptides and cylodipeptides were available for the antimicrobial assay. The strain of *C. truncatum* was isolated in Argentina as one of the most dangerous pathogens of soybean inducing anthracnose symptoms with severe epidemics and expressive yield losses.^44^

All the lipodepsipeptides (tolaasins and WLIP) were produced, purified, and identified as reported in detail in the Materials and Methods section. The two derivatives of tolaasin I and the methyl ester of WLIP were prepared and characterized as reported in detail in the same section and in the Supporting Information. In particular, the ^1^HNMR spectrum (Figure S2, SI) of the hexacetyl derivative of tolaasin I (**15**) essentially differed from that of tolassin I (Figure S1, SI), recorded under the same conditions for the singlets of five acetyl groups in the range of δ 2.20–1.99. Its ESIMS (+), spectrum showed the sodiated adduct ion [M + Na]^+^ at *m*/*z* 2260. The ^1^H NMR spectrum (Figure S4, SI) of the tetrahydro derivative of tolaasin I (**16**) essentially differed from that of tolaasin I, recorded under the same conditions, for the absence of olefinic protons. Its ESIMS (+) spectrum showed the protonated adduct ion [M + H]^+^ at *m*/*z* 1990. Finally, the ^1^H NMR spectrum of WLIP methyl ester (**17**) (Figure S6, SI) essentially differed from that of WLIP (Figure S7, SI), recorded under the same conditions, for the presence as a singlet at δ 3.70 due to the ester methyl group. Its ESIMS (+) spectrum exhibited the sodiated [M + Na]^+^ and the protonated [M + H]^+^ adduct ions at *m*/*z* 1275 and 1289, respectively.

**Figure 4:**
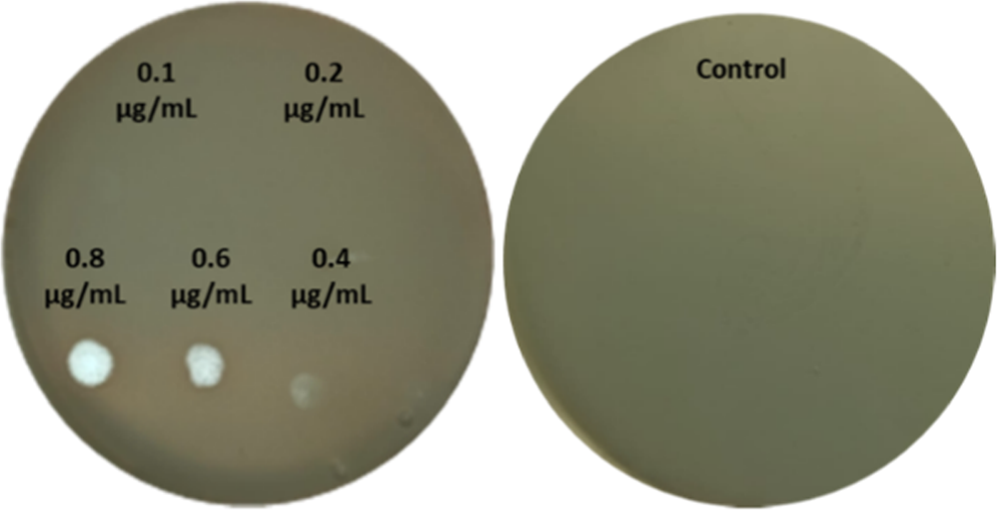
Representative photographs of minimum inhibitory concentration of tolaasin II against the *Pseudomonassyringae* pv. *syringae* strain B475.

**Figure 5:**
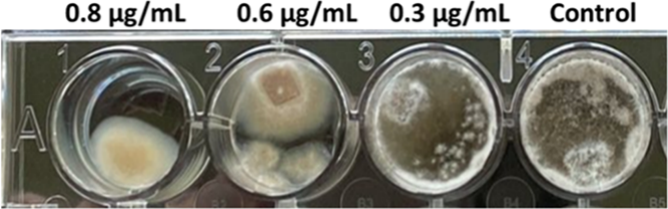
Representative photographs of minimum inhibitory concentration of tolaasin I against the *C. truncatum* strain 17-5-5.

In the first experiment, the lipodepsipeptide tolaasins I, II, D, and E (**1**, **2**, **7**, and **8**, Figure 1) and WLIP (**3**, Figure 2) and their derivatives hexacetyl- and tetrahydro-tolaasin I and WLIP methyl ester (**15**, **16**, and **17**, Figure 2) were assayed against all the plant pathogenic and nonpathogenic bacteria and the fungus *C. truncatum* reported above using antimicrobial and antifungal tests (Figures 4 and 5). The results obtained, summarized in Table 1, showed that among the tolaasins and their two derivatives, the compounds **1**, **2**, and **7** and the tetrahydro tolaasin I (**16**) inhibited all the bacteria and the fungus tested with a MIC in the range of 0.1–0.9 μg/mL. Just for the bacteria *E. coli,* the growth was not inhibited. Tolaasin E and the hexacetyl tolaasin I (**8** and **15**) did not show activity against the three laboratory bacterial strains of *B. subtilis*, *B. megaterium*, and *E. coli*. However, compounds **8** and **15** showed a MIC in the range of 3–6 and 0.7–1 μg/mL, respectively, against the pathogenic bacteria and *C. truncatum*. Furthermore, the sensitivity among the bacteria seems similar, while the fungus appeared to be always less sensitive. The highest antimicrobial activity was shown by tolaasin D (**7**) with a MIC range of 0.1–0.2 μg/mL, and the lesser toxicity was shown by tolaasin E (**8**) and by the two derivatives of tolaasin I (**15** and **16**) with a MIC range of 0.7–1 and 0.2–3 μg/mL, respectively. Comparing the very similar activity of tolaasins I and II (**1** and **2**) seemed that the amino acid residue at the 16 position of the macrolactone ring is not important for the activity as it is l-homoserine (l-Hse) in **1** and l-serine (l-Ser) in **2**. l-Hse is also present at the same position in tolaasin D (**7**); thus, the increased activity showed by the latter, with respect to **1** and **2**, could be due to the presence of a different amino acid residue at the 15 position, which is l-leucine (l-Leu) in **7** and l-*iso*-leucine (l-Ile) in the other two. However, the presence in the same lipodepsipeptide of l-Leu and l-Ser at 15 and 16 positions, respectively, probably induces a noteworthy decrease in antimicrobial activity as observed in tolaasin E (**8**). The acetylation of the hydroxyl group of the fatty acid, l-Ser, l-Hse, and the primary amino groups of d-2,4-diamino butyric acid (d-Dab) and l-lysine (l-Lys) at positions 17 and 18 of macrocyclic lactone and the hydrogenation of two residue 2-butenylbutiric acid (ΔBut) located at 1 and 13 positions of the linear peptide chain, compared to **1**, significantly induced a decrease in activity.

**Table 1:** Minimal Inhibitory Concentration of the Lipodepsipeptides Tested^a^

		MIC (μg/mL)							
ID	strain	WLIP	WILP methyl ester	hexacetyl tolaasin I (**15**)	tolaasin D (**7**)	tolaasin E (**8**)	tetrahydro tolaasin I (**16**)	tolaasin I (**1**)	tolaasin II (**2**)
NCPPB 349	*Pseudomonas caryophylli*	-	-	0.9	0.1	3	0.9	0.2	0.4
ICMP3955	*Pseudomonas syringae* pv. *panici*	-	-	0.7	0.1	3	0.9	0.2	0.4
ICMP2706	*Pseudomonassyringae* pv. *tabaci*	-	-	0.8	0.1	4	0.8	0.3	0.4
B475	*Pseudomonassyringae* pv. *syringae*	-	-	0.8	0.1	3	0.8	0.3	0.4
ICMP6305	*Pseudomonassyringae* pv. *japonica*	-	-	0.7	0.1	4	0.9	0.3	0.4
PY79	*Bacillus subtilis*	0.3	0.5	-	0.2	-	0.2	0.3	0.4
QMB	*Bacillus megaterium*	0.3	0.5	-	0.2	-	0.2	0.3	0.4
DH5α	*Escherichia coli*	-	-	-	-	-	-	-	-
17-5-5	*Colletotrichum truncatum*	3	5	1	0.2	6	3	0.6	0.8

a **-:** no activity.

The nonapeptide WLIP (**3**) that differed from tolaasins for all the three moieties such as the fatty acid, the linear side peptide chain, and the macrocyclic lactone practically did not inhibit the growth of all pathogenic bacteria, while it exhibited activity against the two laboratory Gram-positive strains *B. subtilis* and *B. megaterium*. Despite the lesser activity of tolassins I, II, and D (**1**, **2**, and **7**), WLIP showed antifungal activity against *C. truncatum* with a MIC of 3 μg/mL. Its methyl ester gave very similar activity, suggesting that a lethal methabolism^45^ could work by hydrolyzing the methyl ester group under physiological conditions.

In a second experiment, using the same bioassay method and the same microorganisms, the antimicrobial activity of the 2,5-diketopiperazines, namely cyclo(l-Pro-l-Tyr), cyclo(l-Pro-l-Val), cyclo(L-Pro-L-Leu), and cyclo(D-Pro-L-Tyr) (**9**, **11**, **13**, and **14**), was tested. The results of the bioassay, listed in Table 2, showed that all the dicyclopeptides showed activity against all the bacteria except compound **9** on *E. coli*. The 2,5-diketopiperazine **11** was not toxic. Among the active compounds **9**, **13**, and **14**, the highest antimicrobial activity was showed by maculosin-1 (cyclo(l-Pro-l-Tyr, **9**)) with a MIC range of 15–20 μg/mL. The other two compounds (**13** and **14**) were less active, showing for the pathogenic bacteria and the fungus a MIC range of 500–800 μg/mL, but were more active against the laboratory bacterial strains. The antimicrobial activity of compound **9** is in agreement with its antifungal activity previously reported.^25,26^ The lack of activity of compound **14** also demonstrated that the configuration d or l of the amino acids that constitute the dicyclopeptide is a very important feature to impart activity. In fact, dicyclopeptides **9** and **14** differed only for the opposite d stereochemistry of proline residue in the second one, and its activity is reduced with respect to that of **9** by 50–60 times. The amino acids which constitute the dicyclopeptides also affect the activity as compound **13**, which differs from compound **9** for the substitution of l-Tyr with l-Leu, showing a noteworthy reduction of activity by 40–50 times. Very surprising is the inactivity of dicyclopeptide **11** as recently it showed, among the 2,5-diketopiperazines reported above, the highest activity against *R. fascians*.^27^

**Table 2:** Minimal Inhibitory Concentration of the Cyclodipeptides Tested^a^

		MIC (μg/mL)			
ID	strain	L-Pro-L-Tyr (**9**)	D-Pro-L-Tyr (**14**)	L-Pro-L-Leu (**13**)	L-Pro-L-Val (**11**)
NCPPB 349	*Pseudomonas caryophylli*	15	800	500	-
ICMP3955	*Pseudomonas syringae* pv. *panici*	15	900	700	-
ICMP2706	*Pseudomonassyringae* pv. *tabaci*	15	800	500	-
B475	*Pseudomonassyringae* pv. *syringae*	15	800	600	-
ICMP6305	*Pseudomonassyringae* pv. *japonica*	15	800	600	-
PY79	*Bacillus subtilis*	20	35	15	-
QMB	*Bacillus megaterium*	20	30	30	-
DH5α	*Escherichia coli*	-	20	300	-
17-5-5	*Colletotrichum truncatum*	20	800	500	-

a **-:** no activity.

In conclusion, in testing the antibacterial and antifungal activity, lipodepsipeptides showed growth inhibitory activity 56–60 times higher than that of dicylopeptides. Among the lipodepsipeptides, the nonapeptides such as WLIP, tested on phytopathogenic bacteria and fungus, showed only weaker fungicide activity against *C. truncatum*. In lipodepsipeptides having a longer peptide side chain, the presence of some amino acid residues of the lactone ring is important to increase the activity as was the effect on the activity of tolaasin D for the presence of l-Ile instead of l-Leu residue. The derivatization of their amino acid residues of both the macrocyclic lactone ring and linear peptide side chain weakly affects inhibitory activity. Finally, for tolaasin D, considering the possibility of its large-scale production using a fermenter, a suitable bioformulation could have a potential for practical application as a bacteriocide and fungicide in agriculture and in particular against the pathogens of important agrarian plants that have developed resistance to the common chemical pesticides.

## Supporting Information Available

^1^H NMR spectrum of tolaasin I in CD_3_OD at 500 MHz (Figure S1); ^1^H NMR spectrum of hexacetyltolaasin I in CD_3_OD at 500 MHz (Figure S2); ESIMS spectrum of hexacetyltolaasin I (Figure S3); ^1^H NMR spectrum of tetrahydrotolaasin I in CD_3_OD at 500 MHz (Figure S4); ESIMS spectrum of tetrahydrotolaasin I (Figure S5); ^1^H NMR spectrum of WLIP in CD_3_OD at 500 MHz (Figure S6); ^1^H NMR spectrum of WLIP methyl ester in CD_3_OD at 500 MHz (Figure S7); and ESIMS spectrum of WLIP methyl ester (Figure S8) (PDF)
