# Supplementary material for: Bacterial Lipodepsipeptides and Some of Their Derivatives and Cyclic Dipeptides as Potential Agents for Biocontrol of Pathogenic Bacteria and Fungi of Agrarian Plants
Source: J Agric Food Chem. 2022 Apr 8;70(15):4591–8. doi: 10.1021/acs.jafc.1c08139 (PMC9026286; doi:10.1021/acs.jafc.1c08139)
Supplement: Supplementary file 1 — jf1c08139_si_001.pdf [file jf1c08139_si_001.pdf]

Supporting Information

Bacterial Lipodepsipeptides, Some their Derivatives and Cyclic Dipeptides as Potential Agents to Biocontrol Pathogenic Bacteria and Fungi of Agrarian Plants

Stefany Castaldi,[‖] Alessio Cimmino,[‖] Marco Masi, Antonio Evidente*

Index

Figure S1. ^{1}H NMR spectrum of tolaasin I in CD_3OD at 500 MHz

Figure S2. ^{1}H NMR spectrum of hexacetyltolaasin I in CD_3OD at 500 MHz

Figure S3. ESIMS spectrum of hexacetyltolaasin I

Figure S4. ^{1}H NMR spectrum of tetrahydrotolaasin I in CD_3OD at 500 MHz

Figure S5. ESIMS spectrum of tetrahydrotolaasin I

Figure S6. ^{1}H NMR spectrum of WLIP in CD_3OD at 500 MHz

Figure S7. ^{1}H NMR spectrum of WLIP methyl ester in CD_3OD at 500 MHz

Figure S8. ESIMS spectrum of WLIP methyl ester

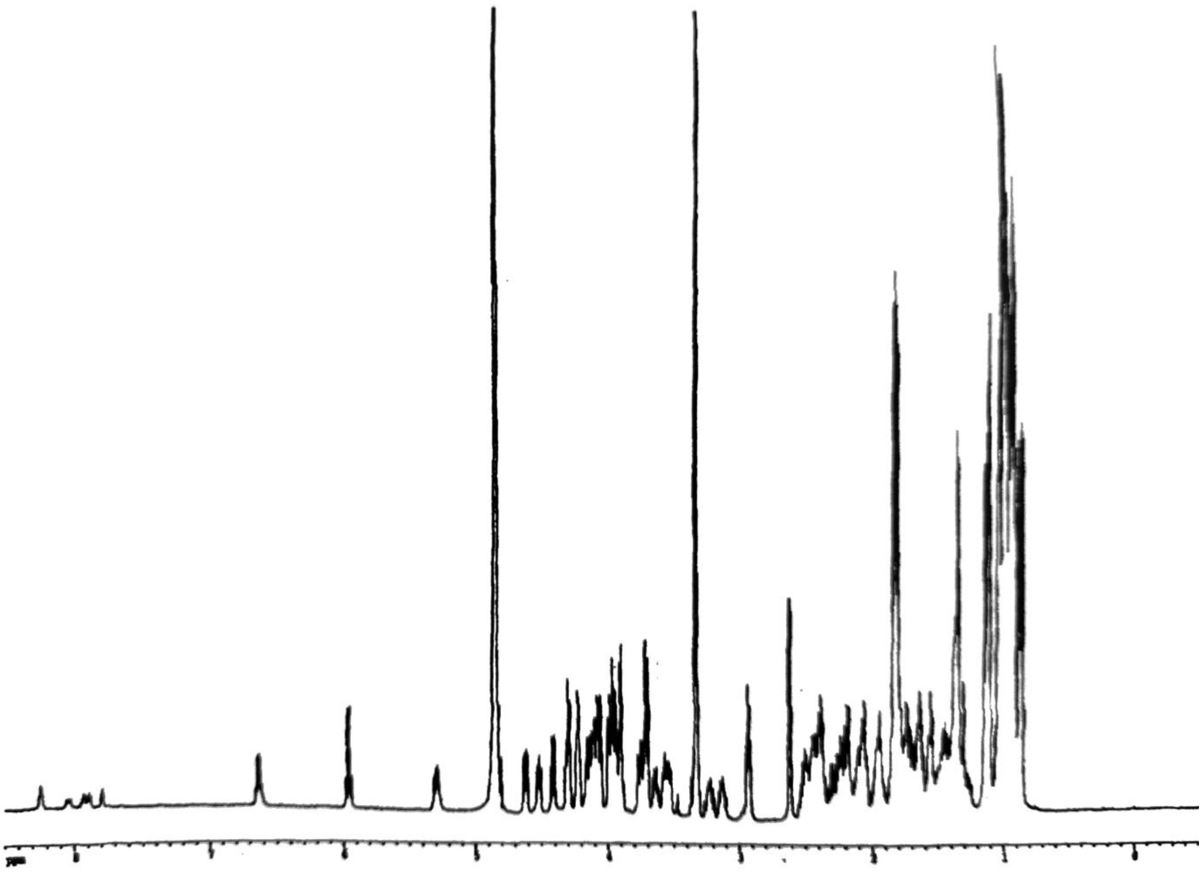

Figure S1. ^{1}H NMR spectrum of tolaasin I in CD_3OD at 500 MHz.

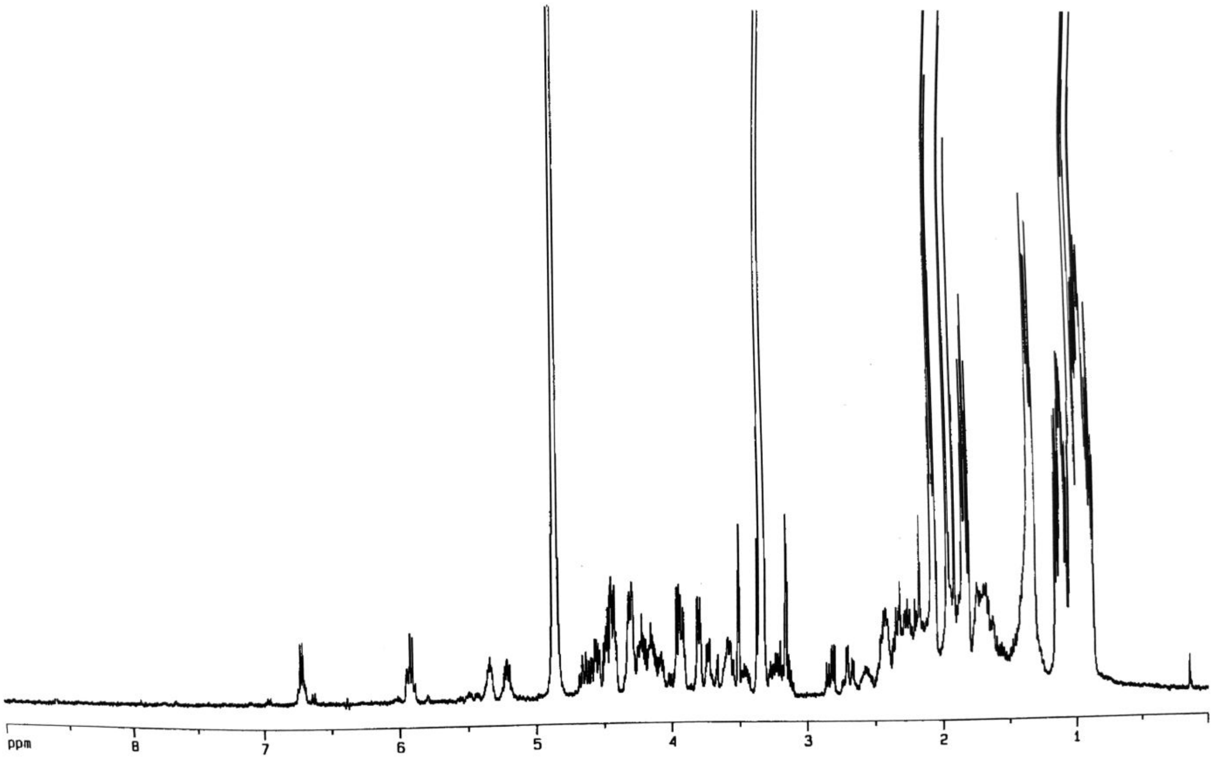

Figure S2. ^{1}H NMR spectrum of hexacetyltolaasin I in CD_3OD at 500 MHz.

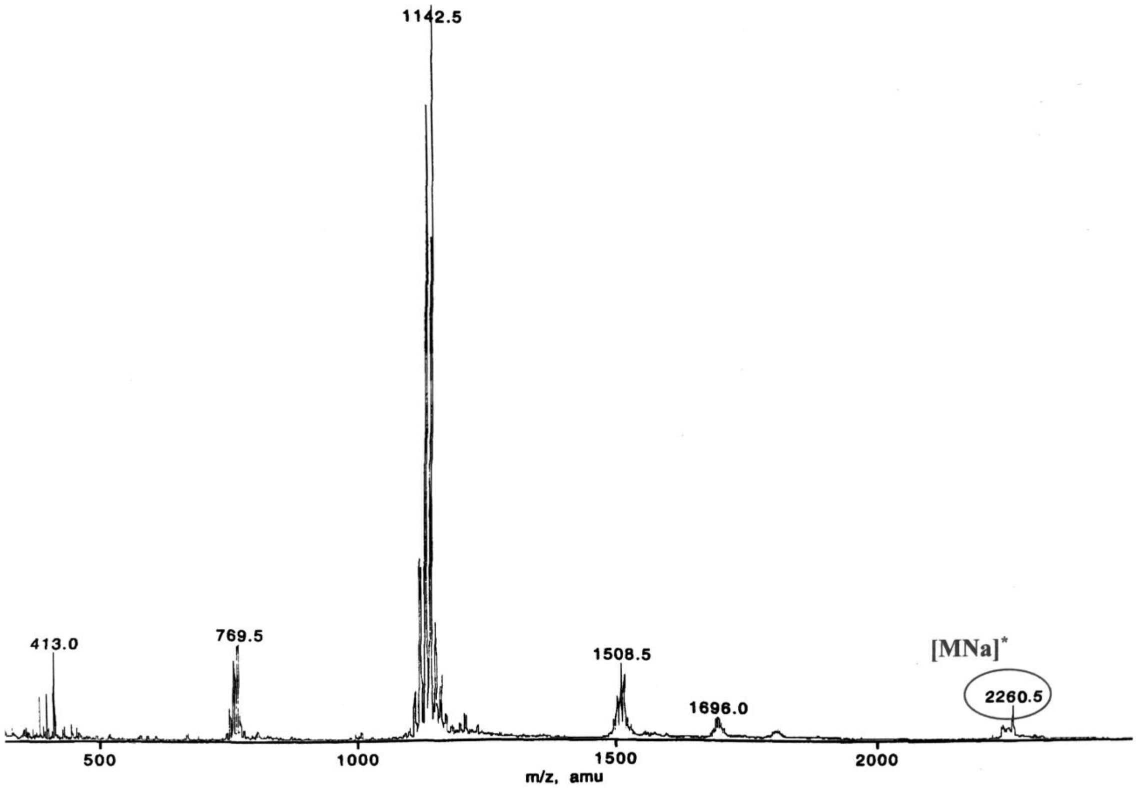

Figure S3. ESIMS spectrum of hexacetyltolaasin I.

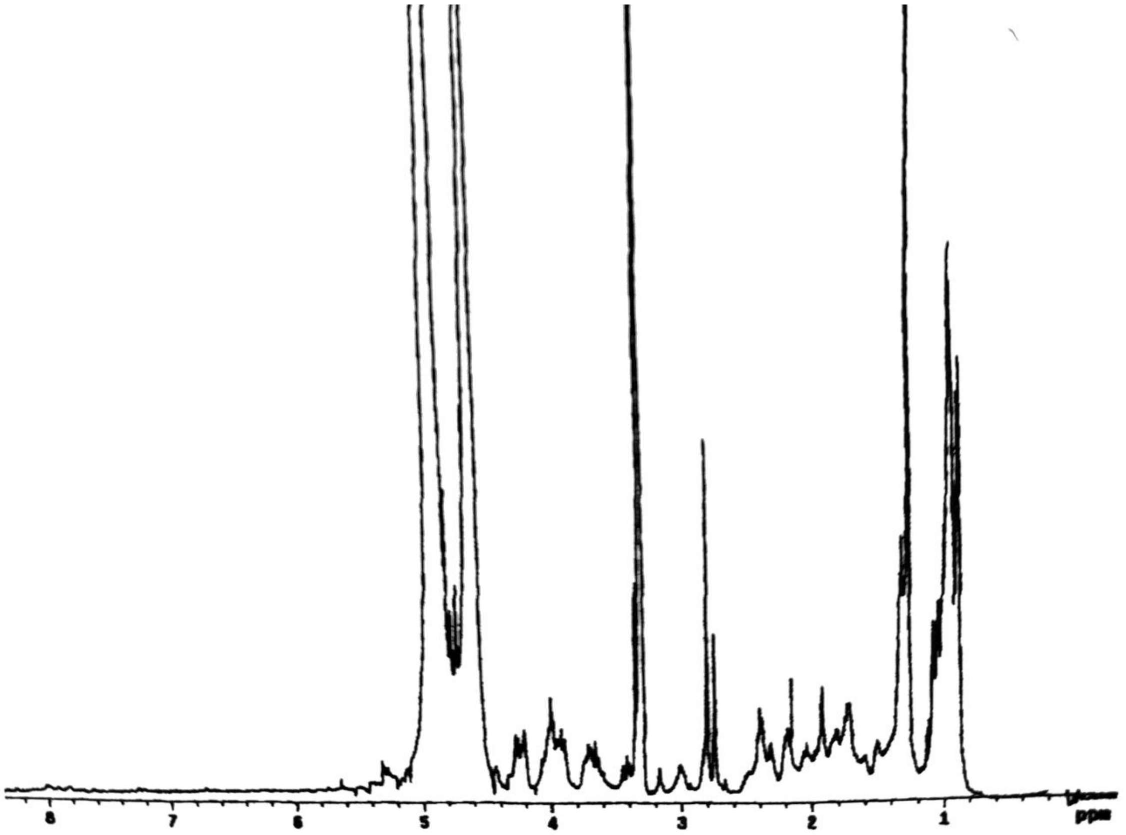

Figure S4. ^{1}H NMR spectrum of tetrahydrotolaasin I in CD_3OD at 500 MHz.

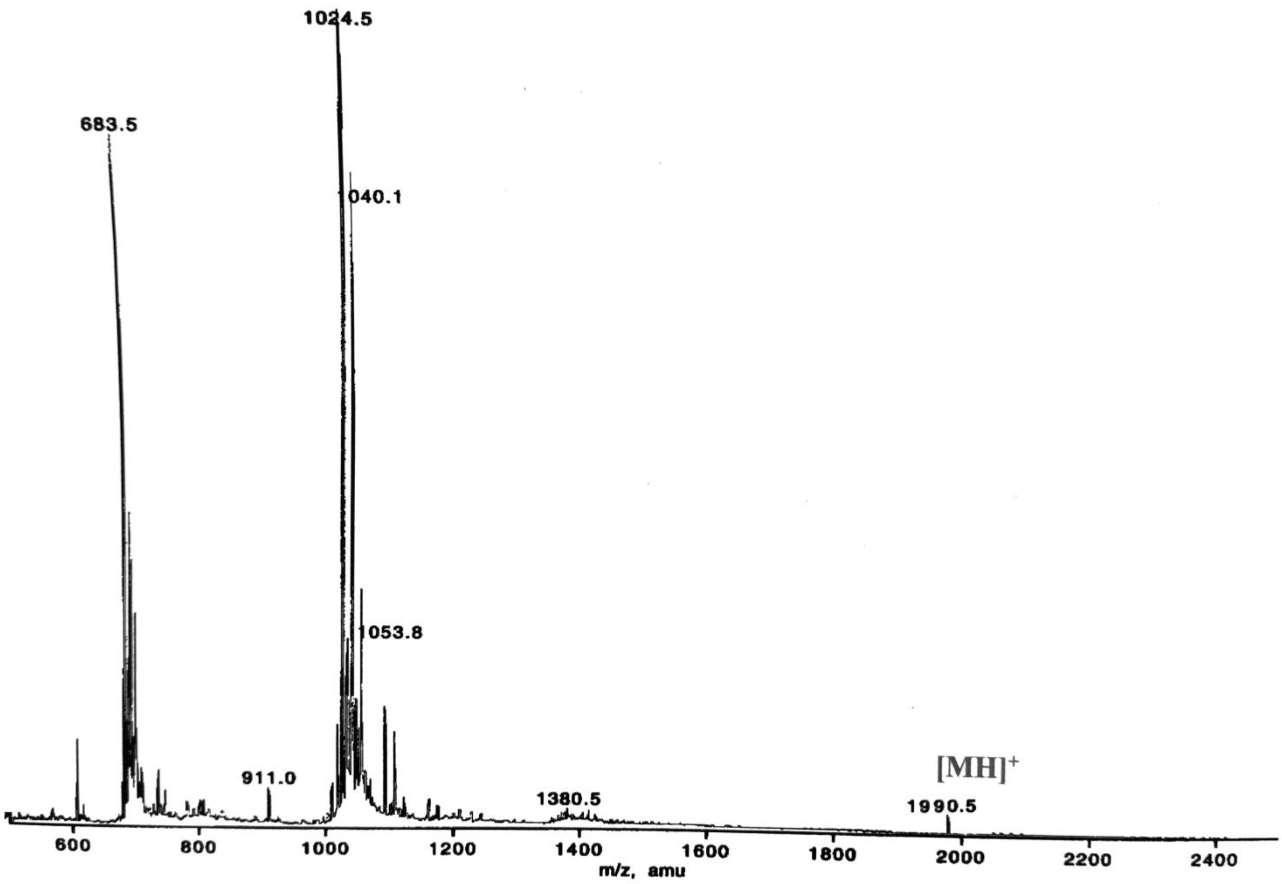

Figure S5. ESIMS spectrum of tetrahydrotolaasin I.

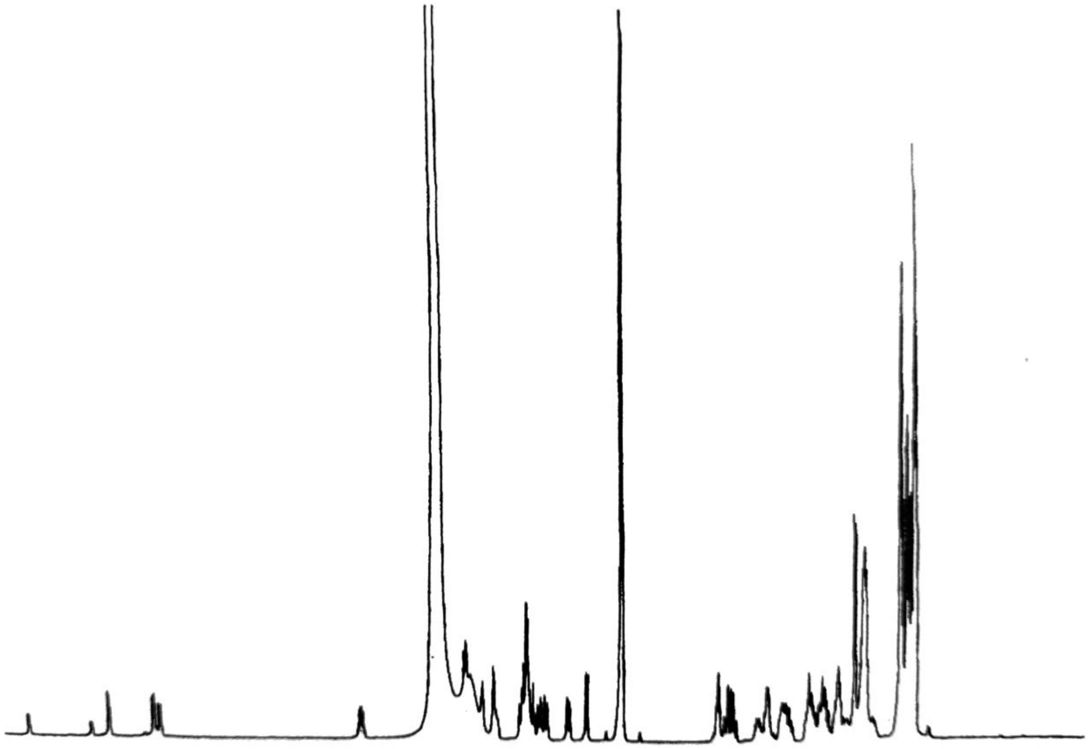

Figure S6. ^{1}H NMR spectrum of WLIP in CD_3OD at 500 MHz.

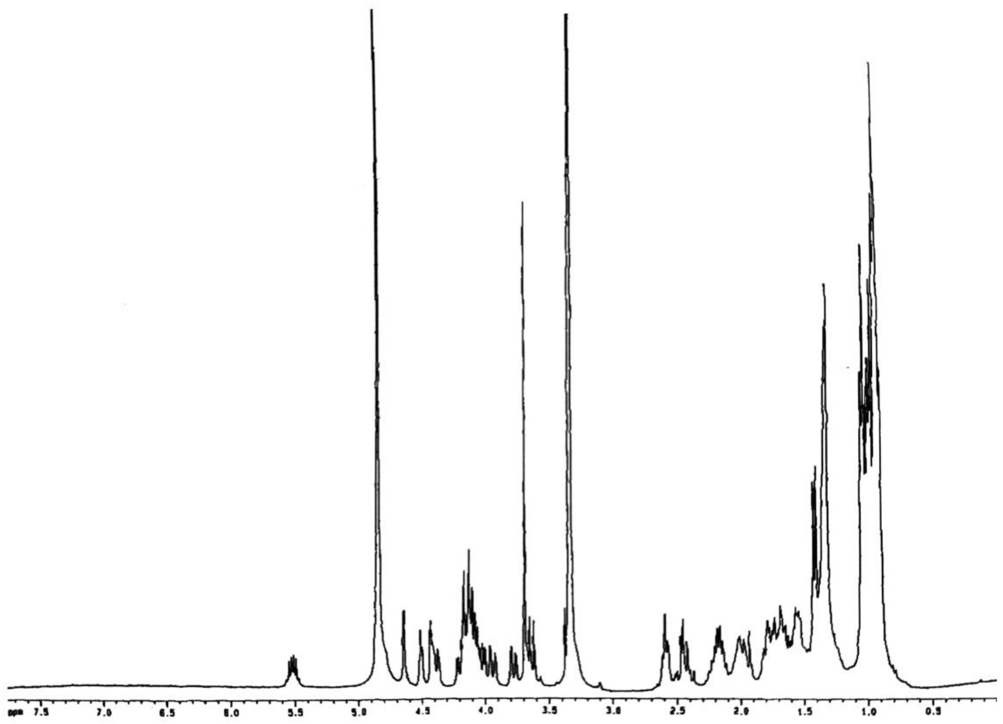

Figure S7. ^1H NMR spectrum of WLIP methyl ester in CD_3OD at 500 MHz.

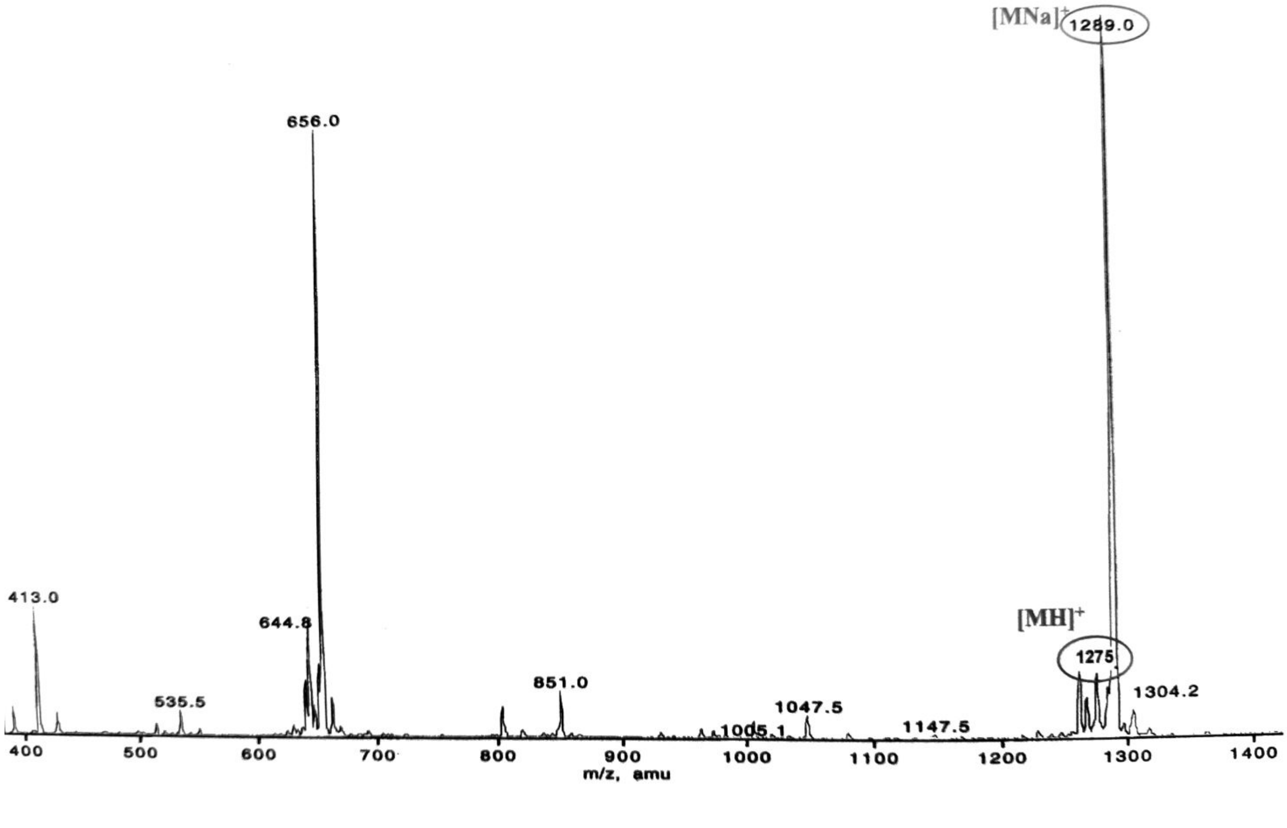

Figure S8. ESIMS spectrum of WLIP methyl ester.
